# Development and validation of an ELISA using a protein encoded by ORF2 antigenic domain of porcine circovirus type 2

**DOI:** 10.1186/1743-422X-7-274

**Published:** 2010-10-19

**Authors:** Shi-Qi Sun, Hui-Chen Guo, De-Hui Sun, Shuang-Hui Yin, You-Jun Shang, Xue-Peng Cai, Xiang-Tao Liu

**Affiliations:** 1 State Key Laboratory of Veterinary Etiological Biology, Key Laboratory of Animal Virology of Ministry of Agriculture, Lanzhou Veterinary Research Institute, Chinese Academy of Agricultural Sciences, Xujiaping 1, Lanzhou, 730046, The People's Republic of China

## Abstract

**Background:**

The capsid protein (ORF2) is a major structural protein of porcine circovirus type 2 (PCV2). A simple and reliable diagnostic method based on ORF2 protein immunoreactivity would serve as a valuable diagnostic method for detecting serum antibodies to PCV2 and monitoring PCV infection. Here, we reported an indirect enzyme-linked immunosorbent assay (I-ELISA) by using an antigenic domain (113-147AA) of ORF2-encoded antigen, expressed in *E. coli*, for diagnosis of PCV infection.

**Results:**

The ELISA was performed on 288 serum samples collected from different porcine herds and compared with an indirect immunofluorescent assay (IFA). In total, 262 of 288 samples were positive as indicated by both I-ELISA and IFA. The specificity and sensitivity of I-ELISA were 87.7% and 93.57%.

**Conclusions:**

This ELISA is suitable for detection and discrimination of PCV2 infection in both SPF and farm antisera.

## Background

Porcine circovirus (PCV) is a member of *circoviridae*. It is a small non-enveloped DNA virus with a circular single-stranded genome [1]. Genomic analysis revealed that there are two distinct genotypes of PCV [2-5]. The PCV1 was identified as a persistent non-cytopathic contaminant of the porcine kidney cell line PK-15 [6,7]. In contrast, PCV2 is considered the primary causative agent for post weaning multisystemic wasting syndrome (PMWS) [8-11]. The genome DNA of both PCV1 and PCV2 consist of several major open reading frames; of these, ORF1, ORF2, and ORF3 have been studied. The ORF1 encodes a replication-associated protein of 35.7 kDa [12], while ORF2 encodes a major immunogenic capsid protein of approximately 30 kDa [13] and ORF3 plays a major role in PCV2-induced apoptosis [14].

Post weaning multisystemic wasting syndrome is a disease of growing pigs that causes low morbidity but high case mortality. The disease is characterized by progressive weight loss, respiratory and digestive disorders, lymphohistiocytics, and lymphoid depletion [8,15,16]. Most regions of the world have reported PMWS cases [5,9,17-23], and it is currently considered an important swine disease with potentially serious economic impacts for the global swine industry.

As a control measure, specific serologic detection is essential. To date, immunoperoxidase monolayer assay (IPMA)[24] and indirect immunofluorescent assay (IFA)[25] are the most widely used diagnostic methods for detecting PCV infection. However, these methods are labor-intensive and time consuming, and carry the risk of virus contamination. These techniques require experienced technicians who can judge the staining reactions accurately. In contrast, enzyme linked immunosorbent assay (ELISA) can decrease the potential bias that may occur with IFA and IPMA and is amenable to automation, so it is suitable for large-scale diagnostics.

Recently, several ELISAs for detecting PCV infection have been developed. Some have been based on cell-culture-propagated PCV2 and specific PCV2 monoclonal antibodies [26]. These assays are more expensive, of greater technical difficulty than ELISA based on recombinant major capsid protein [13]. Recent studied have adopted ELISA based on recombinant major capsid protein expressed in recombinant baculovirus-infected cells [27,28]; however this is still not optimal because it is more difficult to isolate sufficient proteins from this expression system than from bacterial expression systems.

Several antigenic epitopes of the capsid protein were demonstrated at amino acid residues 65-87, 113-147, 157-183, and 193-207. The 113-147 epitope proved to be the immunorelevant epitope for virus type discrimination [29]. Truong et al. [30] developed a peptide-ELISA using a chemically synthesized epitope of PCV2 ORF2. Here, we describe a PCV2 ORF2 immunorelevant epitope (ORF2-E) isolated from a bacterial expression system and used as the coating antigen for ELISA. The aim was to establish an ELISA diagnosis method to detect anti-PCV2 antibody in infected swine.

## Results

### Cloning and sequencing of PCV2 ORF2

**Figure 1:**
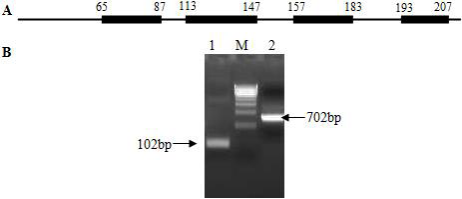
**(A) The map of dominant immunoreactive areas of ORF2**. The amino acid residues of each area are identified. (B) The ORF2 fragment that spans from amino acid 113 to 147 was amplified with a pair of ORF2 primers (Lane 1). The entire ORF2 fragment was used as a positive control (Lane 2). The DNA marker is a 500 bp DNA ladder.

There are five dominant immunoreactive areas on PCV-encoded proteins, one located on ORF1 and four on ORF2 [29]. However, only one antigenic domain (113-147) of ORF2 protein was suitable for an ELISA to detect swine PCV2 infection. We cloned the 102 bp nucleotide encoding the 113-147 peptide of ORF2 protein (Figure 1).

### Analysis of recombinant protein

**Figure 2:**
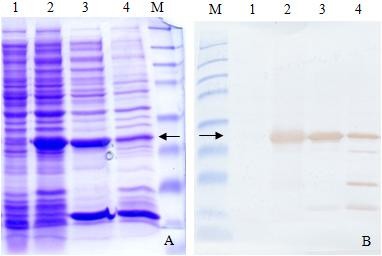
**The expression of GST-ORF2-E protein was analyzed by SDS-PAGE (A) and Western-blotting (B) with an anti-GST monoclonal antibody**. Lane 1, BL21 cell lysate before induction of IPTG; Lane 2, BL21 cell lysate after induction of IPTG; Lane 3, Supernatant of cell lysate after sonication and centrifugation; Lane 4, Pellet of cell lysate after sonication and centrifugation, There was a clear band of 29 kDa (arrow) after induction. The protein marker includes 8 bands at 175, 83, 62, 47.5, 32.5, 25, 16.5, and 6.5 kDa.

We constructed an expression vector, pGEX-ORF2-E, which allowed the ORF2 antigenic domain to be expressed as a GST-tagged fusion protein (GST-ORF2-E) for efficient purification. SDS-polyacrylamide gel electrophoresis (SDS-PAGE) and Western-blotting were used to confirm expression of the recombinant protein. The presence of the fusion protein in the bacterial cell fractions before induction and after induction was analyzed. There was a band of about 29 kDa on the SDS-PAGE gel (Figure 2), both from the sonicated pellet and a more intense band from the supernatant remaining from centrifugation of the sonicated cell suspension (Figure 2), indicating that most of GST-ORF2-E protein was soluble. Western-blotting using the anti-GST monoclonal antibody further confirmed that the fusion protein GST-ORF2-E was expressed correctly in bacterium.

**Figure 3:**
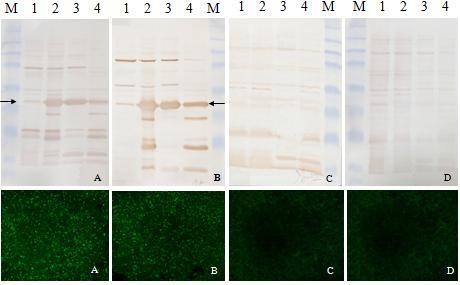
**Western-blotting analysis of the expressed recombinant GST-ORF2-E protein with porcine serum (above) was confirmed by IFA (below)**. A clear band with the expected molecular weight appeared on the NC membrane after incubation with two positive porcine serum samples (A, B), but no equal band appeared when incubated in two samples of negative porcine serum (C, D). Lane 1, BL21 cell lysate before induction of IPTG; Lane 2, BL21 cell lysate after induction of IPTG; Lane 3, Supernatant of cell lysate after sonication and centrifugation; Lane 4, Pellet of cell lysate after sonication and centrifugation; Protein marker includes 8 bands of 175, 83, 62, 47.5, 32.5, 25, 16.5, and 6.5 kDa.

**Figure 4:**
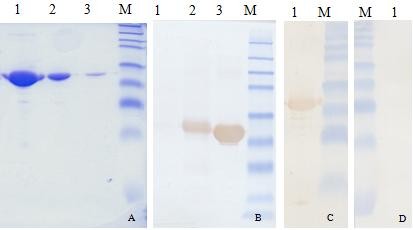
**Confirmation of purified GST-ORF2-E protein by SDS-PAGE and western-blotting**. (A) SDS-PAGE of purified protein after elution. Lane 1: The first elution; Lane 2: The second elution; Lane 3: The third elution. (B) Western-blotting with GST monoclonal antibody. Lane 1, BL21 cell lysate before induction of IPTG; Lane 2, BL21 cell lysate after induction of IPTG; Lane 3, Purified protein. (C) and (D) are results of western-blotting using positive (C) or negative (D) porcine serum as the primary antibody. Protein marker includes 8 bands at 175, 83, 62, 47.5, 32.5, 25, 16.5, and 6.5 kDa.

To test the antigenicity of GST-ORF2-E, we used the PCV2 swine serum as a primary antibody in western-blotting (Figure 3 and Figure 4). There was a strong signal on the NC membrane against positive serum but no signal against negative serum. Similarly, the expected 29 kDa band appeared on the western-blotting membrane using an anti-GST monoclonal antibody and porcine serum.

### Evaluation of GST-ORF2-E proteins ELISA

To coat plates for ELISA, the optimum concentration of antigen was determined by checkerboard titration. A final protein concentration of 0.5 μg/mL was determined. Using this optimal concentration of coating antigen, the optimal dilution of the HRP-conjugated anti-pig IgG was obtained at 1:3000 by checkerboard titration. A field serum dilution of 1 to 100 was selected as an optimum dilution for assays. Phosphate buffered saline containing 0.1% Tween-20 and 5% (w/v) non-fat dry milk as the blocking buffer, and PBS containing 0.1% Tween-20 and 1% (w/v) non-fat dry milk as the dilution buffer, were determined to have a good positive/negative (P/N) ratio.

**Figure 5:**
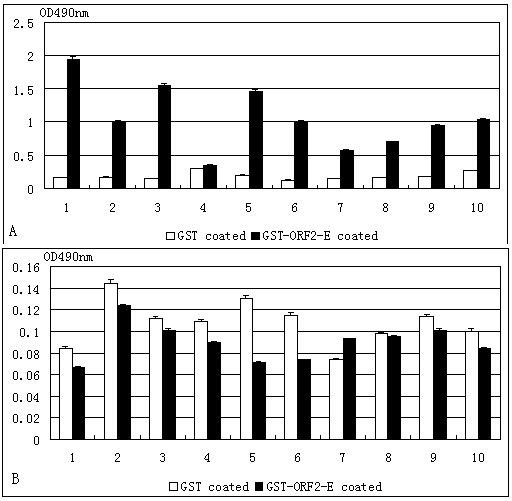
**ELISA using GST as a reference for evaluation of non-specific binding**. Twenty serum samples including 10 positive sera (A) and 10 negative sera (B) were used. Each serum sample was run in quadruplicate, two on GST-ORF2-E antigen and two on GST antigen wells. Positive and negative control sera were induced in every plate.

To determine whether the GST tag interfered with the GST-ORF2-E ELISA, we coated plates with either purified GST protein or GST-ORF2-E and tested the optical density (OD) after treatment with 10 samples of positive and 10 samples of negative sera (Figure 5). The statistical analysis (two-sample paired *T*-test) showed that average OD of positive sera tested by GST-ORF2-E was significantly different than that tested on GST alone (*P *< 0.01) and the average OD of negative sera tested by GST-ORF2-E was significant different than that tested on GST alone (0.01 <*P *< 0.05). Moreover, the average OD of positive sera tested on GST alone was not significant different from that of negative sera tested on GST alone (*P *> 0.05).

### Confirmation of negative-positive cutoff

A cutoff point for each assay was determined so that DSN and DSP were maximized while the sum of false negative and false positive results was minimized. The OD at 490 nm for negative sera ranged from 0.068 to 0.209. The averaged OD of 25 negative pig sera in the ELISA was 0.12466, yielding a suitable cut-off OD value of 0.224313 (mean + 3SD) in this assay and indicated that 99% of the negative sera have OD values below 0.22. The positive threshold was set at 0.22. Based on this criterion, all 25 positive sera have OD values above 0.22.

### Evaluation of assay repeatability

The repeatability test was done by comparing OD ratios of triplicate results from each field serum sample tested in the same plate (intra-assay repeatability) or in different plates at different times (inter-assay repeatability). The intra-assay CV of 10 positive serum samples ranged from 0.12% to 14.87%, with a median value of 2.34%, while those of negative serum samples ranged from 0.46% to 6.45%, with a median value of 2.17%. The inter-assay CV for positive serum samples was between 11.26% and 37.04%, with a median value of 19.03%, whereas the CV for negative serum samples was between 10.16% and 38.26%, with a median value of 31.74%. These data showed that the assay was repeatable and yielded a low and acceptable variation.

### Evaluation of assay specificity and sensitivity

The PCV2 GST-ORF2-E ELISA results were obtained from 288 serum samples. The results for these serum samples were compared with those obtained by the IFA reference method (serum sample diluted 1:50). The diagnostic sensitivity and specificity of the ELISA test were determined using the formulae given in the methods. The result demonstrated that the sensitivity and specificity of the ELISA test were higher than IFA (Table 1). The negative and positive serum determinations were 8 and 280 by IFA and 25 and 263 by ELISA. The specificity relative to IFA was 87.7% and sensitivity was 93.57% (the agreement rate was 93.4%). Cross-reaction was analyzed by testing the reactivity of antibodies against other porcine viruses with the antigenic domain antigen. As showed in Table 2, there was no cross-reactivity between the PCV2 113-147 domain of ORF2 and antibodies against other porcine viruses, proving that the domain antigen was specific for antibody to PCV2.

**Table 1:** Comparison between the IFA and ELISA for field sera

		ELISA	
		
	Result	Negative	Positive
IFA	negative	2.43%(7/288)	0.347%(1/288)

IFA	positive	6.25%(18/288)	90.97%(262/288)

**Table 2:** Cross-reaction analysis of the domain based ELISA to antisera against other swine viruses

Antisera to	OD value (mean ± 3SD)
PCV2	1.313 ± 0.125

CSFV	0.028 ± 0.004

PPV	0.030 ± 0.006

PRRSV	0.025 ± 0.003

Non-infected	0.023 ± 0.008

### Evaluation of correlation between ELISA and IFA

**Figure 6:**
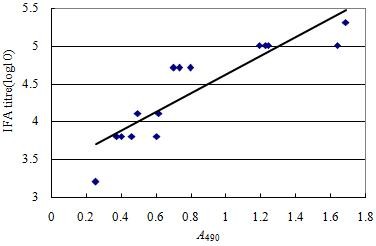
**Scatter plots of log10 IFA titers of 16 serum samples against OD ratios of the corresponding serum obtained from GST-ORF2-E ELISA**.

The correlation between IFA titer and OD ratio was determined by plotting endpoint IFA titers of 16 serum samples with different levels of antibodies to PCV2 against OD ratios of the corresponding serum (Figure 6). The results indicated that the linear relationships between log10 titer of IFA and OD ratio obtained from GST-ORF2-E ELISA (spearman's rank correlation = 0.9665; *P *< 0.0001) were similar, which means the relationships between IFA titers and OD ratios of GST-ORF2-E are linear (the regression equation was: IFA titer = 1.21339 × A490 + 3.41189, r^2 ^= 0.7897, *P *< 0.001). In conclusion, OD ratio obtained from GST-ORF2-E ELISA could be used to predict IFA titer.

## Discussion

The ORF1 and ORF2 of both PCV types show about 60 to 80 percent sequence identity at the amino acid level, and this homology was shown to be relativity well conserved between different PCV isolates [4,5,12]. This indicates that there will be significant antigenic cross-reactivity between viral products of the PCV genotypes. Even though currently available methods, such as indirect immunoperoxidase and immunofluorescence assays, are widely used for the serological diagnosis of PCV2 infection, these assays are labor intensive and time consuming. Furthermore, cross reactions between PV1 and PV2 could lead to false-positives. It was previously shown that there is common immunoreactivity epitope on the ORF1-encoded protein, but there was no cross-reactivity between the ORF2-encoded proteins of PCV1 and PCV2 [9,25,29]. Therefore, in order to develop a PCV2-specific indirect ELISA diagnosis assay, we first focused on the expression of whole ORF2 in *E. coli *that bares an arginine-enriched nuclear localization signal. Liu et al. [31] previously reported that the whole ORF2 protein was not expressed successfully in *E. coli*., so we designed a vector containing only the immunorelevant epitope [29] of ORF2 protein in frame with a GST tag to efficiently isolate protein from bacteria (about 20 mg/L cells). In addition, the GST tag increased the solubility of target proteins, and does not generally interfere with biological activity. The recombinant GST-ORF2-E protein reacted strongly with PCV2-infected swine serum, demonstrating its biological activity and also suggesting possible use in diagnostic assays. The result in this study proved that affinity-purified GST-ORF2-E protein can be employed to improve the sensitivity and specificity of the I-ELISA.

To determine whether the GST tag in recombinant ORF2 protein enhanced the OD value to produce false-positive results, we compared plates coated with GST protein alone with plates coated with GST-ORF2-E protein. According to average OD value from positive or negative serum, GST tag in recombinant GST-ORF2-E was not specific to swine serum, demonstrating that GST-ORF2-E can be used as a coating antigen for the detection of PCV-2 antibodies by indirect ELISA.

The newly developed ELISA showed repeatability for negative sera as indicted by the low variability among replicates from the same sample. There was smaller differences between intra-assay trials than inter-assay trials, however, suggesting that optimization is not complete, especially the stability of antigen. However, the CV for positive and negative serum samples in two assays indicated that the intra-assay variability of this GST-ORF2-E ELISA was acceptable.

The OD ratio of the GST-ORF2-E ELISA showed significant agreement with the antibody rates of IFA for field sera, so the ELISA can be used for direct comparison of antibody concentrations in field samples and could be of particular importance for dynamic studies of PCV. However, several IFA-positive sera were classified as negative by GST-ORF2-E ELISA. This may be due to antibody binding affinity and stability of the antigen-antibody complex in the short peptide relative to binding onto the whole virus. Indeed, the source of antigen for IFA was fixed cells, while the ELISA antigens were soluble. So, as expected, both types of antigens contain shared and distinct epitopes which will be recognized by different antibodies. Another reason may be that the PCV1 contamination maybe results in significant false-positive in IFA. Moreover, as Nawagitgul et al. reported [13], evaluating a newly developed assay by comparison with a widely used assay is not an absolute standard of comparison. In this study, sera with an IFA titer of 1:50 or more were defined as positive, while for the ELISA, sera with 1:100 or more were considered positive. Therefore, it is possible that the IFA might result in more false positives due to the low dilution of serum samples. However, the GST-ORF2-E ELISA is specific for PCV2, which is related to the PCV2 specific antigenic epitope in ELISA. This result also confirmed that the GST-ORF2-E ELISA can be used to selectively detect the anti-PCV2 antibody in infected swine.

## Conclusions

The present study clearly shows that detection of PCV2 antibodies by I-ELISA using ORF2-E as an antigen is specific, sensitive, inexpensive, rapid, and easy to perform. Moreover, the method can distinguish PCV2-infected pig sera from PCV1-infected serum. Consequently, the I-ELISA described in this report may be a particularly valuable test for the routine diagnosis of PCV2 infection in pigs.

## Methods

### Cell virus and sera

The permanent PK15 cell line, which was free of PCV, was maintained in minimal essential medium (MEM) supplemented with 10% fetal bovine serum (FBS) (Gibco BRL). The wild-type PCV2 virus used in the study was originally isolated from a kidney tissue sample of a pig with naturally occurring PMWS. A total of 288 field serum samples were collected from different region of Gansu province, China. Positive sera against classic swine fever virus (CSFV), porcine parvovirus (PPV), and porcine reproductive and respiratory syndrome virus (PRRSV) from SPF pigs were purchased from the Chinese Institute of Veterinary Drug Control.

### Cloning and sequencing of PCV2 capsid protein antigenic domain

The PCV2 genome was used as template for amplification of the virus capsid protein gene by polymerase chain reaction (PCR). The PCR was performed using a pair of primers (ORF2-EF:5'-GC GGA TCC CAG GGT GAC AGG GGA GTG GGC T-3' and ORF2-ER:5'-GC CTC GAG TTA GCG GGA GGA GTA GTT TAC A-3'). The thermocycle condition was an initial denaturing at 94°C for 2 min, followed by 30 cycles of 94°C for 20 sec, 60°C for 20 sec, and 72°C for 30 sec. The elongation time was 8 min at 72°C. The PCR fragment was cloned between the BamHI and XhoI sites of the pGEX-4T-1 vector (Amersham-Pharmacia Biotech) and in frame with the glutathione S-transferase (GST) sequence. The nucleotide sequence of the construct was verified by DNA sequencing.

### Expression and purification of ORF2-E fusion proteins in E. coli

Recombinant GST-ORF2-E protein and GST protein were expressed in *E. coli *BL21. *E. coli *containing the expression plasmid were grown overnight at 37°C in LB medium with 100 μg/mL ampicillin. Cells were then diluted 1:100 and allowed to grow at 37°C to an optical density between 0.6 and 0.8 at 600 nm. Isopropylthio-β-D-galactoside (IPTG) was added to a final concentration of 0.1 mM. Following 3 h of growth, cells were harvested by centrifugation.

The GST-ORF2-E fusion protein was purified from the bacterial lysate by using a glutathione affinity column (Amersham-Pharmacia Biotech). Briefly, cell pellets were resuspended in ice-cold PBS and sonicated for 10 min (power 3, on 30 sec; off 30 sec). After the sonicated solution was centrifuged, the supernatant was then transferred to a 50% slurry of Glutathione Sepharose 4B equilibrated with PBS. Followed incubation with gentle agitation at room temperature for 30 min, the matrix was transferred to a disposable Column. The matrix was washed with PBS and the fusion protein eluted by glutathione elution buffer. The eluate was collected and GST fusion protein was analyzed by SDS-PAGE and Western-blotting.

### Protein expression analysis

Proteins were separated by SDS-PAGE on 12% acrylamide gels using a discontinuous buffer system. For Western blotting, proteins were transferred to nitrocellulose membranes (GIBCO BRL) in transfer buffer (20 mM Tris-HCl, 190 mM glycine, 20% methanol, pH 8.3) using a Mini Trans-blot transfer system (Bio-Rad) at 100 V for 1 h. The membranes were blocked with 5% nonfat dried milk in TTBS (Tris-buffered saline containing 0.05% Tween-20) at room temperature for 1 h and then incubated with anti-GST monoclonal antibody (Dako, 1:500) or swine sera (1:200) at room temperature for 1 h. After three washes in TTBS, the membranes were incubated with 1:2000 peroxidase-conjugated anti-mouse or anti-swine antibody (Dako) at room temperature for another 1 h. After washing with TTBS, the reacted patterns were visualized with DAB (3, 3'-Diaminobenzidine) substrate (Sigma).

### IFA

To prepare plates for IFA, the PK-15 cells were split one day before infection. A 100 μL suspension of freshly trypsinized PK-15 cells at a concentration of 5×10^4 ^cells/mL was transferred into a 96-well plate. The PCV2 at a multiplicity of infection (MOI) of 0.1 were inoculated into rows 1, 3, 5 and 7 of the 96-well plate. Mock-infected PK-15 cells were prepared similarly to PCV2-infected cells and seeded in alternate rows. Cells were treated with 300 mM D-glucosamine in Hank's buffer at 37°C for 20-30 min at 4-6 hours post-infected (hpi) and then cultured in a humidified incubator aerated with 5% CO_2 _for 72 h at 37°C. Cells were fixed with 4% PFA (polyformaldehyde) in PBS at room temperature for 30 min and washed with PBST (PBS pH 7.4 containing 0.1% Tween-20). The cells were then incubated for 10 min at room temperature with 0.1% Triton X-100 in PBS, followed by incubation for a further 1 h at 37°C with pig serum diluted 50 times in PBST containing 5% FBS. After three washes with PBST, cells were stained for 1 h at 37°C with FITC-conjugated rabbit anti-swine IgG (Dako) diluted 100 times in PBST containing 5% FBS. After washing, plates were examined using fluorescence microscopy.

### ELISA procedure

Ninety-six microtiter plates (Nunc Maxisorp) were coated with 100 μL GST-ORF2-E antigen in 0.05 M bicarbonate buffer (pH 9.6) and incubated overnight. After two washes in PBST, the plates were blocked with 100 μL PBST containing 5% non-fat dry milk for 1 h at 37°C. After washing, a diluted pig serum with PBST containing 1% non-fat dry milk was added, and plates were again incubated for 1 h at 37°C. After rinsing three times with PBST, 100 μL diluted rabbit anti-swine IgG conjugated with peroxidase (Dako) in the PBST containing 1% non-fat dry milk was added, and then incubated at 37°C for another 1 h. The plates were then washed three times, and the colorimetric reaction was developed using 50 μL substrate solution (FAST ο-phenylenediamine dihydrochloride, Sigma) for 15 min at 37°C. Color development was stopped with 50 μL of 2N H_2_SO_4_, and optical density (OD) was read at 490 nm.

### Confirmation of negative-positive cutoff

The negative-positive cutoff value was set by the average OD ratio of 25 field negative sera and 25 positive sera by GST-ORF2-E ELISA. A negative-positive threshold for each assay was calculated using the Microsoft Excel spreadsheet.

### Evaluation of assay repeatability

Ten negative serum samples and 10 positive serum samples were selected for the repeatability test. For intra-assay (within-plate) repeatability, three replicates of the same serum sample were performed in the same plate. For inter-assay (between-run) repeatability, three replicates of each sample were run in different plates on different occasions. Mean OD ratio; standard deviation (SD), and coefficient of variation (CV) of three replicates of each test were calculated.

### Evaluation of assay specificity and sensitivity

The diagnostic sensitivity (DSn) and specificity (DSp) of the ELISA test were determined using the following formulae: DSn = TP/(TP+FN)×100 (where TP is the true positive and FN is the false negative) and DSp = TN/(TP+TN) ×100 (where TN is true negative and FP is false positive). The accuracy is (TP+TN)/total number of serum samples tested ×100 [13].

### Evaluation of correlation between ELISA and IFA

ELISA values (OD ratios) obtained from sera taken from the sixteen PCV2-infected pigs were compared with antibody titers determined by IFA on PCV2-infected cells. The IFA was performed on serial dilutions of the corresponding sera from 1:50 to 1:51,200. A correlation between the IFA titer and the OD ratio was determined by the Spearman's correlation coefficient.

## Competing interests

The authors declare that they have no competing interests.

## Authors' contributions

SQS conceived and designed the study, organized protocol developments, interpreted of data and wrote the manuscript. HCG took part in development of ELISA and IFA protocols, carried out ELISA and IFA, contributed to the interpretation of the findings and revised the manuscript. DHS, SHY and YJS carried out PCR and protein expression and purification. XTL and XPC additionally contributed to the study design, contributed to conception, interpretation of data and revision of the manuscript. All authors' have read and approved the final manuscript.
